# The complete mitochondrial genome of *Conus quercinus* (Neogastropoda: Conidae)

**DOI:** 10.1080/23802359.2018.1501314

**Published:** 2018-08-17

**Authors:** Po-Wei Chen, Wen-Lung Wu, Deng-Fwu Hwang

**Affiliations:** a Department of Food Science, National Taiwan Ocean University, Keelung, Taiwan;; b Biodiversity Research Center, Academia Sinica, Taipei, Taiwan

**Keywords:** *Conus quercinus*, worm-hunting sea snails, mitochondrial genome, next-generation sequencing

## Abstract

The complete mitochondrial genome sequence of cone snail *Conus quercinus* a kind of worm-hunting sea snails, was performed by next-generation sequencing. The mitogenome is 16,439 bp in length, including 13 protein-coding genes, 22 tRNA genes, two ribosomal RNA genes (*12S* and *16S rRNA*), and one control region. It has overall base composition of A (28.1%), T (38.2%), C (14.7%), and G (18.6%). It shows 75.9% identity with *C. capitaneus*, which also belongs to worm-hunting sea snail. The phylogenetic analysis was conducted with 22 closely related species to assess their phylogenetic relationship. The complete mitogenome of the *C. quercinus* provides important DNA molecular data for further phylogeography.

Cone snails are common names for a large group of venomous predatory sea snails and marine gastropod molluscs. There are over 600 species of cone snails classified under four kinds of genus, including *Conus, Conasprella, Profundiconus*, and *Californiconus* (Puillandre et al. 2015). All of them are in one family, the Conidae. Based on their prey preference, cone snails can be divided into three groups, piscivorous, molluscivorous, and vermivorous (Olivera 1997; Le Gall et al. 1999). There are approximately 30 records of humans killed by cone snails. Human victims suffer little pain, because the venom contains an analgesic component (Nelson 2004).

*Conus quercinus *(Lightfoot 1786), a kind of vermivorous (worm-hunting) sea snail, also names the oak cone (Carpenter and Niem 1998). Their sizes are between 60 mm and 140 mm (Röckel et al. 1995). This species usually distribute in the Indo-West Pacific, from East Africa to eastern Polynesia; north to Japan and Hawaii, and south to Queensland and New Caledonia. They usually bury in the sand during the day but actively foraging for food during evening (Carpenter and Niem 1998).

The specimens of *C. quercinus* (voucher no. 20150421-029; with GenBank accession no. MH400188) in this study were collected from Penghu, Taiwan (23.565N, 119.576E). The samples were deposited in Marine Toxins Lab., Department of Food Science, National Taiwan Ocean University, Taiwan. The total genomic DNA was extracted from muscle using magnetic bead technique with the KingFisher magnetic processors (ThermoFisher Scientific Inc., Worcester, MA). The raw next-generation sequencing reads generated from MiSeq sequencer (Illumina, San Diego, CA) were *de novo* assembled and reference mapping was conducted by commercial software (Geneious V11, Auckland, New Zealand) to produce a single circular form of complete mitogenome with about an average 45.2 coverage (3114 out of 11,798,800 reads, 0.026%). The complete mitochondrial genome of *C. quercinus* is 16,439 bp in size, including 13 protein-coding genes, 22 tRNA genes, two ribosomal RNA genes (*12S* and *16S rRNA*), and one control region. The overall base composition of *C. quercinus* is 28.1% for A, 38.2% for T, 14.7% for C, and 18.6% for G. It shows 75.9% identity with *C. capitaneus* (KX155573), which is also a worm-hunting cone snail. The protein coding rRNA and tRNA genes of *C. quercinus* mitogenome were predicted by using MITOS (Bernt et al. 2013) and tRNAscan-SE (Schattner et al. 2005).

We used MEGA 6 (Tamura et al. 2013) to construct the phylogenetic relationships of the *C. quercinus* and related families by neighbour-joining method with 1000 bootstrap replicates based on the 13 protein-coding genes and two ribosomal RNA genes of the other 22 complete mitochondrial genomes of Neogastropoda sea snails, which are reported in GenBank of NCBI database. Bootstrap support values were relatively high, with 10 nodes having values >95%, and nine nodes demonstrating 100% bootstrap support (Figure 1). *C. quercinus* was grouped together with seven other cone snails from the family Conidae. The lineages of Conidae strongly supported in this report and agreed with previous studies (Bouchet et al. 2011; Puillandre et al. 2014).

**Figure 1.**
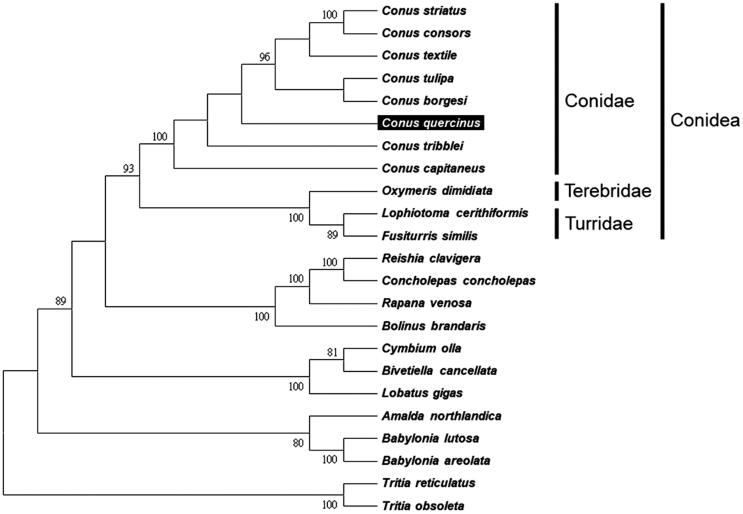
Phylogenetic tree generated using the neighbour-joining method based on complete mitochondrial genomes. *Conus striatus* (KX156937), *C. consors* (KF887950), *C. textile* (DQ862058), *C. tulipa* (KR006970), *C. borgesi* (EU827198), *C. quercinus* (MH400188), *C. tribblei* (KT199301), *C. capitaneus* (KX155573), *Oxymeris dimidiata* (EU827196), *Lophiotoma cerithiformis* (DQ284754), *Fusiturris similis* (EU827197), *Reishia clavigera* (DQ159954), *Concholepas concholepas* (JQ446041), *Rapana venosa* (KM213962), *Bolinus brandaris* (EU827194), *Cymbium olla* (EU827199), *Bivetiella cancellata* (EU827195), *Lobatus gigas* (KM245630), *Amalda northlandica* (GU196685), *Babylonia lutosa* (KF897830), *B. areolata* (HQ416443), *Tritia reticulatas* (EU827201) and *Tritia obsoleta* (DQ238598).
